# The Expression and Potential Role of Tubulin Alpha 1b in Wilms' Tumor

**DOI:** 10.1155/2020/9809347

**Published:** 2020-08-25

**Authors:** Qiong-Qian Xu, Li-Ting Qin, Song-Wu Liang, Peng Chen, Jin-Han Gu, Zhi-Guang Huang, Xia Yang, Li Gao, Shi-Shuo Wang, Yi-Ge Luo, Lin-Le-Yi Liu, Jun Wang, Jin-Yan Lin, Gang Chen, Jia-Bo Chen

**Affiliations:** ^1^Department of Pediatric Surgery, The First Affiliated Hospital of Guangxi Medical University, Nanning 530021, China; ^2^Department of Pathology, The First Affiliated Hospital of Guangxi Medical University, Nanning 530021, China; ^3^Department of First Clinical Medical College, Guangxi Medical University, Nanning 530021, China

## Abstract

We explored the difference in expression of tubulin alpha 1b (TUBA1B) between Wilms' tumor (WT) and normal tissues (NT) from in-house patients and databases, to determine TUBA1B expression in WT and the predictive pathways of coexpressed genes. In-house RNA-sequencing data were performed with WT and NT from three patients from our institute. Other four RNA-sequencing and microarray data were also downloaded from multiple public databases. The TUBA1B expression between WT and NT was analyzed by Student's *t*-test and meta-analysis. The correlation between the expression of TUBA1B and other genes in each study was analyzed. Genes with *p* < 0.05 and *r* > 0.5 were considered as the coexpressing genes of TUBA1B. Overlapping the coexpressed genes of the five studies, including three in-house patients (3 WT *vs.* 3 NT), GTEx-TARGET (126 WT *vs.* 51 NT), GSE2172 (18 WT *vs.* 3 NT), GSE11024 (27 WT *vs.* 12 NT), and GSE73209 (32 WT *vs.* 6 NT), were performed with limma and VennDiagram packages in R software. The website of WEB-based GEne SeT AnaLysis toolkit were used to analyze the gene ontology (GO) and Kyoto Encyclopedia of Genes and Genomes (KEGG) functional annotations for the overlapped genes. The results showed that the relative expression of TUBA1B in WT tissues from in-house three patients was 280.0086, 141.7589, and 303.8292 and that in NT was 16.5836, 104.8141, and 12.79 (3 WT *vs.* 3 NT, *p* = 0.0285, ROC = 100%, SMD = 2.74). Student's *t*-test and meta-analysis in all studies revealed that the expression of TUBA1B was upregulated in WT tissues compared to that in NT (*p* < 0.05, SMD = 2.89, sROC = 0.98). Finally, the research identified the expression of TUBA1B in WT tissues was significantly upregulated than that in NT. The coexpressed genes of TUBA1B were enriched in the pathway of DNA replication, mismatch repair, cell cycle, pathogenic Escherichia coli infection, and spliceosome.

## 1. Introduction

Wilms' tumor (WT), also called nephroblastoma, usually originates from embryonic renal precursor cells in which the cell growth and/or differentiation is dysregulated during development. It accounts for 90% of childhood renal tumors and constitutes 7% of all childhood cancers and is the commonest species of kidney tumor in childhood with an annual incidence of 8–10 per million. It is recognized as an embryonal tumor due to its histological mimicry of stages in nephrogenesis and early age of onset [1–5]. However, some patients still face problems such as a high degree of malignancy, advanced tumor, recurrence, poor prognosis, and treatment-related side effects affecting the quality of life [2]. In recent years, the molecular mechanism of various types of tumor development has gradually become clear. The precise treatment of tumors by molecular-targeted therapy has gradually appeared as a new method. However, the research results of the molecular mechanism of tumors are mainly reflected in the field of adult oncology. The molecular mechanisms underlying the development of solid tumors in children have been investigated to a lesser extent. WT lacks specific tumor markers; therefore, it is even more important to clarify the pathogenesis of malignant tumors in children, to find effective tumor markers, provide new evidence and improvised clinical diagnosis and treatment, and improve the rate of tumors being cured.

Tubulin alpha 1b (TUBA1B), also known as K-ALPHA-1, is a protein-coding gene and a member of the human consensus coding sequence (http://asia.ensembl.org/Homo_sapiens/Gene/Summary?db=core;g=ENSG00000123416;r=12:49127782-49131397). Tubulin is the main constituents of cytoskeleton, and it has five different forms—*α*-, *β*-, *γ*-, *δ*-, and *ε*-tubulin. The *α*- and *β*-tubulin heterodimers that form the microtubules reversibly and dynamically aggregate into microtubules—the cytoskeletal elements that regulate the cell shape, cell adhesion, cell movement, replication and division, and drive mitosis and transport within the cells [6–8]. The *α* and *β*-tubulin heterodimers form polar protofilaments through head-to-tail binding, forming hollow microtubule walls laterally. They are at the core of many aspects of cell biology, acting as orbits for molecular motors and generating forces through their dynamic growth and contraction during the cell cycle [9, 10]. The structure of tubulin provides key information about the mechanism by which it accomplishes its cellular effects and the physiological environment in which these effects are exerted. The occurrence and development of WT is a complex biological process involving multigene participation and regulation, and it is being studied at the molecular level. For example, TUBA1B has been studied in hepatocellular carcinoma and mantle cell lymphoma.

In this study, we used the RNA-sequencing data from in-house WT patients and also downloaded the RNA-sequencing data and microarray from multiple public databases to first explore the TUBA1B expression levels and their possible role in WT patients.

## 2. Materials and Methods

### 2.1. Gene Expression Data

We obtained WT and NT from three patients who underwent surgery for WT at the Department of Pediatric Surgery of the First Affiliated Hospital of Guangxi Medical University between December 2018 and May 2019. Two patients were two years old each, and the third was three years old; all of them were confirmed to have WT by histopathology. The expression of mRNA in the WT and NT was identified using RNA-sequencing by the NovaSeq 6000 platform. In order to further confirm the differences in expression in the WT and NT in the WT patients, the TPM (transcripts per million) expression values from the RNA-sequencing data of 126 WT tissues and 6 NT were downloaded from the Therapeutically Applicable Research to Generate Effective Treatments (TARGET) database (https://ocg.cancer.gov/programs/target); the TPM expression value of the RNA-sequencing data in 45 NT (as supplementary control tissue) were downloaded from the Genotype-Tissue Expression (GTEx) database (https://gtexportal.org/), [11]. The combination of these two databases was called GTEx-TARGET. All TPM expression values were standardized by log2 (*x* + 0.001). We further searched the literature published before February 23, 2020, in the Gene Expression Omnibus (GEO) database (http://www.ncbi.nlm.nih.gov/geo/), [12], Sequence Read Archive (SRA) database (https://www.ncbi.nlm.nih.gov/sra), [13], Oncomine database (https://www.oncomine.org), [14], and ArrayExpress database (https://www.ebi.ac.uk/arrayexpress/), [15]. The search term was “TUBA1B OR Tubulin alpha 1b OR Tubulin-*α*1B OR K-ALPHA-1” and “nephroblastoma OR Wilms' tumor OR Wilm tumor OR embryoma of kidney.” The inclusion criteria were as follows: (i) the object of study is human tissue, and (ii) the expression data for TUBA1B expression in WT and normal kidney tissue must be used to calculate the standardized mean difference (SMD).

### 2.2. Differences in the TUBA1B Expression between WT and NT

Student's *t*-test was performed using the GraphPad 8.0 software to map the violin plots and receiver operating characteristic (ROC) curves to demonstrate the expression of TUBA1B in each study and the ability to distinguish between WT and NT.

To further clarify the expression level of TUBA1B between the WT and NT in the WT patients, we performed a meta-analysis using the Stata 14.0 software. If the heterogeneity analysis was *p* > 0.01 and *I*^2^ < 50% when there was no heterogeneity in statistics, the fixed effects model was used for meta-analysis; otherwise, a random effects model was used for analysis. The mean (M) and standard deviation (SD) value for each study was calculated to obtain the SMD and 95% confidence interval (95% CI). The funnel plot was used to analyze the publication bias, and no significant publication bias was considered when *p* > 0.05. The Midas module was selected for diagnostic meta-analysis, summarizing the receiver operating characteristic (sROC) curve; the forest map of sensibility and specificity was drawn, and the area under the curve (AUC) was calculated to determine the ability of TUBA1B to distinguish between WT and NT. In order to evaluate the stability and reliability of the meta-analysis results, the sensitivity analysis was performed.

### 2.3. The Screening of Coexpressed Genes in TUBA1B and Their Predictive Pathways

**Figure 1:**
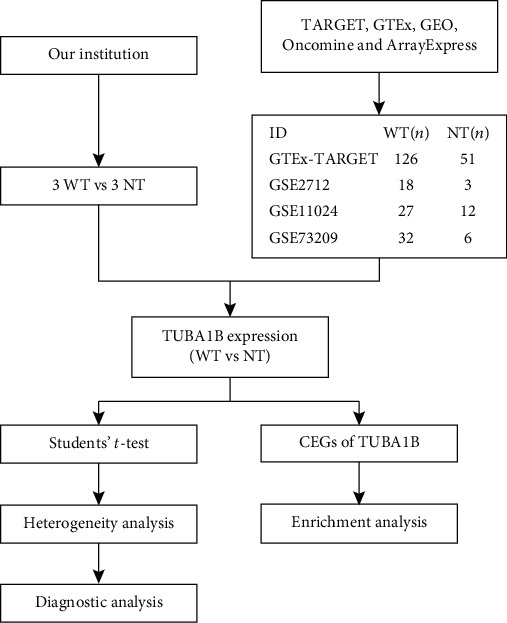
The workflow of this research. *n*: number.

The correlation between the TUBA1B expression and the expression of all the genes in all studies was analyzed. Genes with *p* < 0.05 and *r* > 0.5 were considered to be the coexpressed genes of TUBA1B. The work was performed by R limma and VennDiagram packages [16, 17]. Finally, the overlapping coexpressed genes were calculated. The web-based gene set analysis toolkit (WebGestalt) [18] was used to analyze the Gene Ontology (GO) and Kyoto Encyclopedia of Genes and Genomes (KEGG) database for the presence of overlapping coexpressed genes. The workflow of the study is shown in Figure 1.

## 3. Results

### 3.1. Differences in the TUBA1B Expression

**Figure 2:**
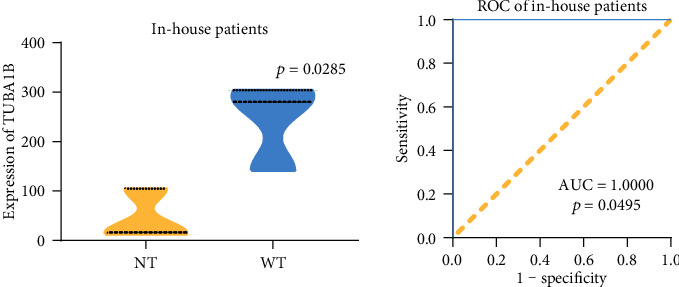
The three WT patients in this study showed higher TUBA1B expression than the NT patients (*p* = 0.0285), and the credibility was high (AUC = 100%, *p* = 0.0495).

**Figure 3:**
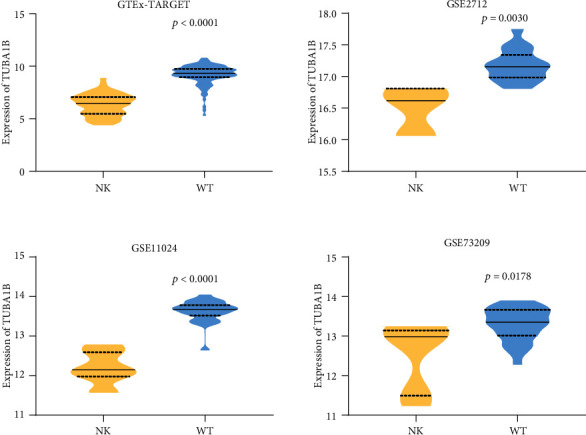
Violin plots for four studies. The four studies suggested that TUBA1B was higher in WT tumor tissues than in NT (*p* < 0.05).

**Figure 4:**
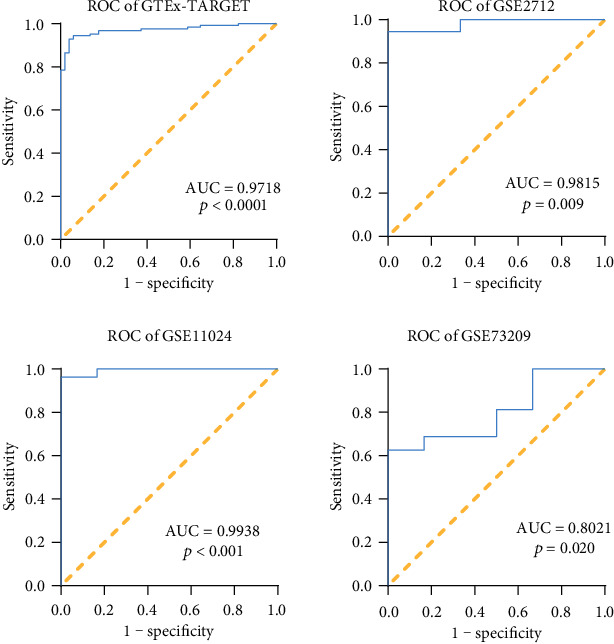
ROC curves for four studies. All of the AUCs in the four studies were more than 80%, and the *p* values were less than 0.05, revealed to be higher.

The relative expression of WT tissues in three in-house patients was 280.0086, 141.7589, and 303.8292 (*M* ± SD = 241.8655667 ± 87.50922111), and that of NT was 16.5836, 104.8141, and 12.79 (*M* ± SD = 44.72923333 ± 52.06958087). Student's *t*-test results in three in-house patients revealed that the TUBA1B expression in the WT tissues was upregulated compared to that in the NT for three patients (3 WT vs. 3 NT, *p* = 0.0285) with an AUC of 100% (Figure 2). For a more comprehensive study, we obtained the TUBA1B expression in 126 WT tissues and 6 NT from the TARGET database. Simultaneously, TUBA1B expression was obtained for 45 NT from the GTEx database. This was for 126 WT tissues and 51 NT samples from GTEx-TARGET database (126 WT vs. 51 NT, *M* ± SD 126 WT = 9.21290037 ± 0.911917003, *M* ± SD 51 NT = 6.33030899 ± 1.042394126). In addition, a total of three eligible WT tissue gene chips were screened out according to the established retrieval strategy, including GSE2712 (18 WT vs. 3 NT, *M* ± SD 18 WT = 17.17291098 ± 0.247686396, *M* ± SD 3 NT = 16.49720674 ± 0.386323037), GSE11024 (27 WT vs. 12 NT, *M* ± SD 27 NT = 13.62791894 ± 0.274908552, *M* ± SD 12 NT = 12.19912535 ± 0.403852992), GSE73209 (32 WT vs. 6 NT, *M* ± SD 32 NT = 13.29800962 ± 0.41302759, *M* ± SD 6 NT = 12.52242602 ± 0.878915291). These gene expression chips were finished based on the chip platforms of GPL10558 Illumina HumanHT-12 V4.0 expression beadchip, GPL96 Affymetrix Human Genome U133A Array, and the GPL6671 Affymetrix GeneChip Human Genome U133 Plus 2.0 Array. Student's *t*-test results of the four datasets showed *p* < 0.0001, *p* = 0.003, *p* < 0.0001, and *p* = 0.0178 (GTEx-TARGET, GSE2712, GSE11024, and GSE73209) (Figure 3). The AUC values were 0.9718, 0.9815, 0.9938, and 0.8021, respectively (Figure 4). All the results indicated that the expression of TUBA1B was higher in the WT tissues than that in the NT.

### 3.2. Meta-Analysis of TUBA1B Expression

**Figure 5:**
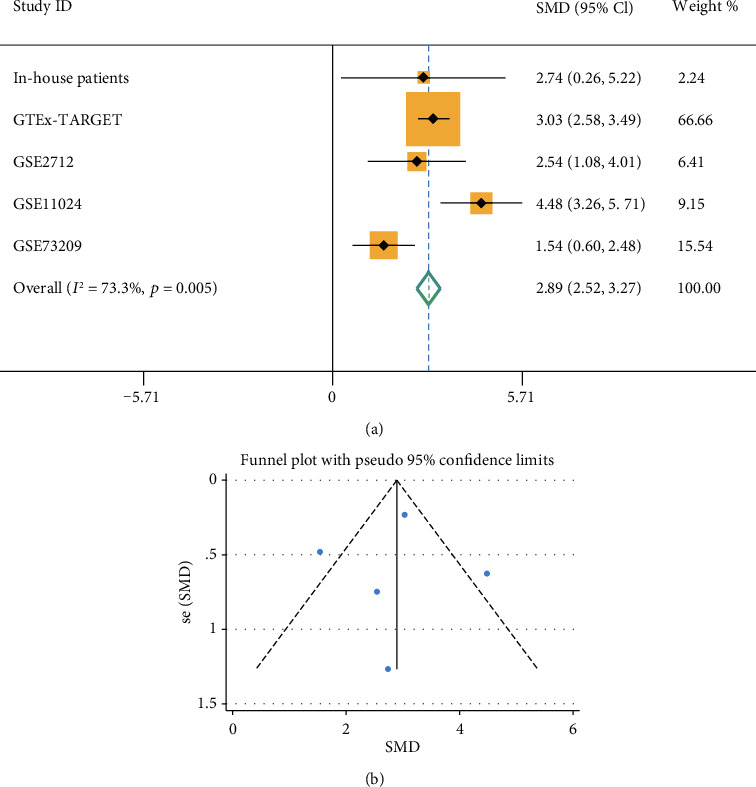
(a) The expression of TUBA1B in WT tissue by meta-analysis. The results showed that *I*^2^ = 73.3%, *p* = 0.005, 95% CI was 2.52-3.27, and SMD was 2.89, overall. (b) Funnel diagram was evaluated for publication bias in meta-analysis. It was found to be basically symmetrical.

**Figure 6:**
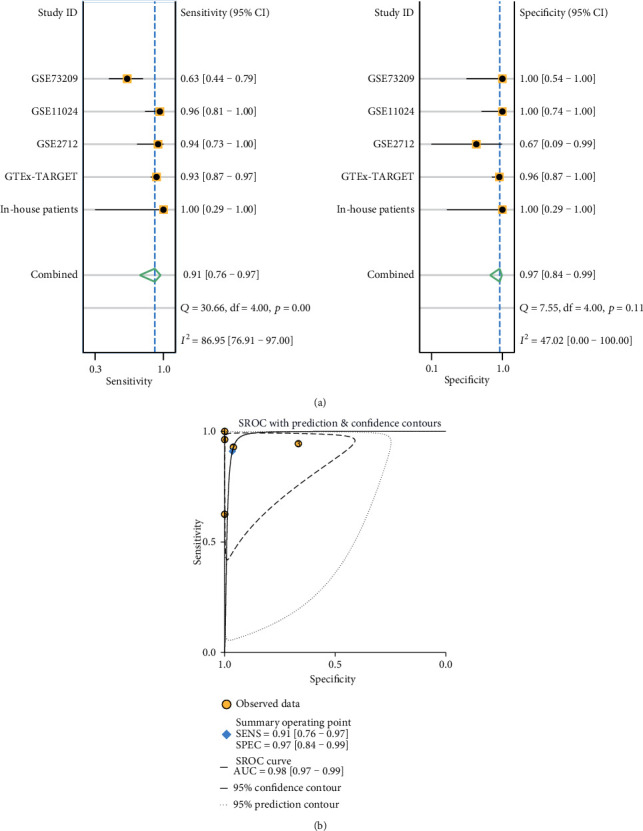
(a) The *Q* test of sensitivity was *p* < 0.001, which indicated that the heterogeneity among the studies was statistically significant, and the *I*^2^ was 86.95%, indicating that the heterogeneity accounted for a large proportion. In addition, the specificity *Q* test (*p* = 0.11) revealed that the heterogeneity among the included studies was not statistically significant. (b) The AUC of sensitivity was 0.91, the AUC of specificity was 0.97, and the AUC of sROC was 0.98, indicating that the research was very credible.

**Figure 7:**
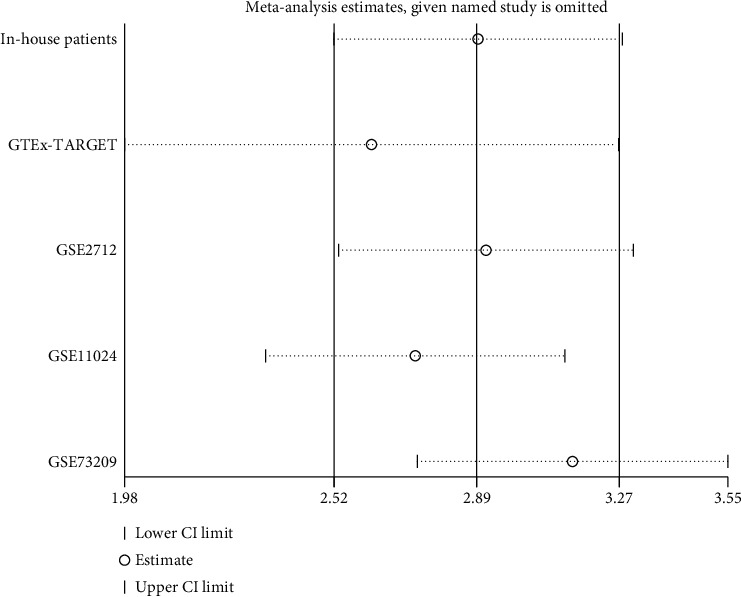
Influence analysis. The results of the influence analysis showed that the elimination of any study did not affect the high expression of TUBA1B in the WT patients.

Based on the five studies, we further verified the expression of TUBA1B in the WT patients by meta-analysis. The results of the heterogeneity analysis showed that *I*^2^ = 73.3% and *p* = 0.005, suggesting that there was great heterogeneity; hence, the random effects model was selected for the meta-analysis. The meta-analysis of five studies revealed TUBA1B expression in WT tissue was higher than that in NT (in-house patients: SMD = 2.74, 95%CI = 0.26-5.22; GTEx-TARGET: SMD = 3.03, 95%CI = 2.58-3.49; GSE73209: SMD = 1.54, 95%CI = 0.60-2.48; GSE2712: SMD = 2.54, 95%CI = 1.08-4.01; GSE11024: SMD = 4.48, 95%CI = 2.52-3.27; overall: SMD = 2.89, 95%CI = 2.52-3.27) (Figure 5(a)). The funnel diagram shows that the figure was basically symmetrical (Figure 5(b)). The heterogeneity in the sensitivity was high, and the heterogeneity of specificity was not statistically significant (Figure 6(a)). The AUC of the sROC curve was 0.98 (Figure 6(b)), which means that the diagnostic test was very accurate. In order to make the published bias evaluation more comprehensive, we carried out the Egger test and Begg's test in five studies and all the results were without an obvious bias (*p* = 0.918, *p* = 1.000). The influence analysis in the five studies showed that the elimination of any study had little impact on the result (Figure 7).

### 3.3. TUBA1B Coexpression Genes and Their Enrichment Analysis

**Figure 8:**
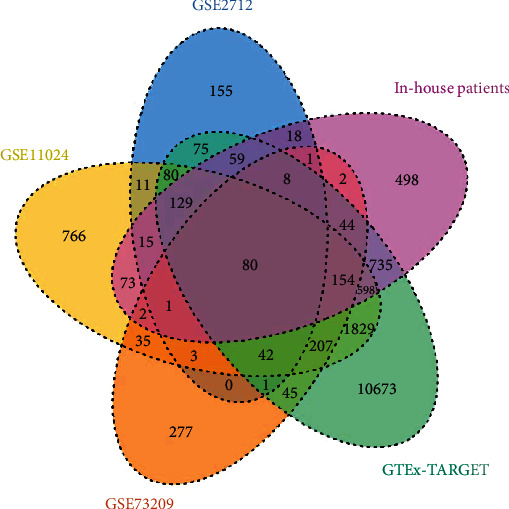
Venn diagram of the five studies. A total of 80 overlapping genes were obtained from in-house patients, GTEx-TARGET, GSE2712, GSE11024, and GSE73209.

**Figure 9:**
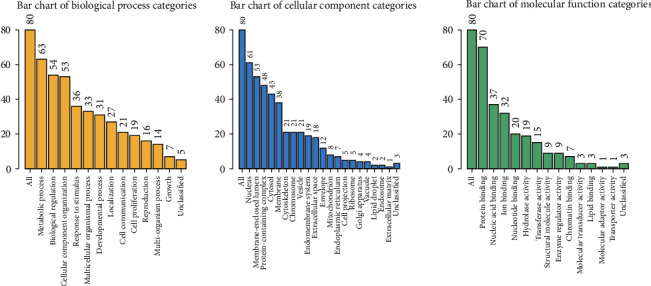
The GO functional annotation for 80 coexpressed genes. The top three BP comments were “metabolic process,” “biological regulation,” and “cellular component organization.” The top three CC comments were the “nucleus,” “membrane-enclosed lumen,” and “protein-containing complex.” The top three MF comments were “protein binding,” “nucleic acid binding,” and “ion binding.”

**Figure 10:**
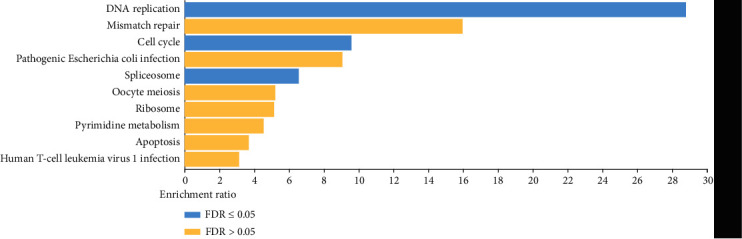
KEGG enrichment analysis for the coexpression of TUBA1B. The top five KEGG enrichment pathways were enriched in “DNA replication,” “mismatch repair,” “cell cycle,” “pathogenic Escherichia coli infection,” and “spliceosome.”

We separately obtained 3632, 677, 4024, 901, and 14759 coexpression genes in the in-house patients, GSE2712, GSE11024, GSE73209, and GTEx-Target. Then, 80 overlapping coexpression genes were obtained from the five studies (Figure 8). Based on these coexpression genes, the top three terms of biological processes (BP) were found to be that of the “metabolic process,” “biological process,” and “cellular component organization.” The top three terms for the cellular component (CC) were the “nucleus,” “membrane-enclosed lumen,” and “protein-containing complex.” The top three terms of molecular function (MF) were “protein binding,” “nucleic acid binding,” and “ion binding” (Figure 9). The top five pathways of KEGG enrichment analysis were DNA replication, mismatch repair, cell cycle, pathogenic Escherichia coli infection, and spliceosome (Figure 10).

## 4. Discussion

In this study, we analyzed the WT and NT of in-house patients (3 WT vs. 3 NT) and demonstrated that the expression of TUBA1B in the WT tissues was higher than that in the NT, and these results were also confirmed by data from multiple public databases including GTEx-TARGET (126 WT vs. 51 NT), GSE2172 (18 WT vs. 3 NT), GSE11024 (27 WT vs. 12 NT), and GSE73209 (32 WT vs. 6 NT). This was the first study to report that the expression of TUBA1B in the WT tissues was higher than that in the NT, indicating that TUBA1B might play an essential role in WT. In order to explore the possible ways in which TUBA1B may influence WT, we obtained 80 coexpression genes and its KEGG enrichment analysis. It was found that coexpression genes were mainly enriched in DNA replication, mismatch repair, cell cycle pathway, etc. (Figure 7).

Tubulin is a major ingredient of the microtubules that combine with two moles of GTP, one each on the *α* and *β* chains. Microtubules are polar filaments constructed from *αβ*-tubulin heterodimers that exhibit a series of structures in vitro and in vivo. Tubulin heterodimers are spirally arranged on the microtubule wall, but many physiologically relevant architectures exhibit a break in the helical symmetry known as the seam [9]. The differential biosynthesis of tubulin is the result of posttranscriptional regulation of the tubulin mRNA. This mechanism, known as tubulin self-regulation, is a negative feedback loop that indirectly adjusts the stability of mature splicing rather than that of the unspliced tubulin pre-mRNA through nonpolymeric tubulin [19–21]; here, the regulation occurs at the mRNA stability level rather than at the transcriptional level [22].

TUBA1B has had been reported in other cancer studies. Cancer cells acquire mitotic drug resistance through beta I-tubulin mutations and alterations in the expression of the beta-tubulin isotypes. *β*-I-tubulin mutations can alter the dynamics of the microtubule assembly and induce resistance to microtubule stabilizers and sensitivity to microtubule stabilizers in the cancer cells [23]. TUBA1B has also been reported in hepatocellular carcinoma and mantle cell lymphoma. TUBA1B was found to be upregulated in the hepatocellular carcinoma tissues and the proliferating hepatocellular carcinoma cells. In addition, increased expression of TUBA1B in patients with hepatocellular carcinoma was associated with poor overall survival and tolerability of paclitaxel [24]. TUBA1B expression was also implicated in the poor prognosis in mantle cell lymphoma [25].

So far no study has shown the relationship between TUBA1B and WT. In this study, we used RNA sequencing data and microarray from in-house three patients (3 WT vs. 3 NT), GTEx-TARGET (126 WT vs. 51 NT), GSE2172 (18 WT vs. 3 NT), GSE11024 (27 WT vs. 12 NT), and GSE73209 (32 WT vs. 6 NT) to demonstrate the upregulation of TUBA1B in the WT tissues. The GO functional comment results suggested that TUBA1B and its coexpression genes may affect the occurrence of WT through signaling pathways such as the metabolic process in BP, nucleus in CC, and the protein binding in MF. The KEGG enrichment analysis revealed that the TUBA1B coexpression genes were significantly expressed in DNA replication, mismatch repair, cell cycle pathway, etc. In the KEGG pathway analysis, we found that DNA replication was the most abundant pathway.

Tumor development is a complex process. It is not yet known whether TUBA1B would have an impact on the WT patient prognosis and ongoing research efforts are required. We confirmed that the expression of TUBA1B in the WT tissue was significantly higher than that in the NT by many methods, but we do not have experimental support for the deficiency in NT. In order to make our research more rigorous, we had intended to conduct cell experiments to verify our results. However, we could not buy the correct WT cell lines in China. The G-401, SK-NEP-1, and WT-CLS1 were incorrect WT cell lines [26, 27]. Hence, we had to terminate this idea.

## 5. Conclusion

The research identified the expression of TUBA1B in WT tissues was significantly upregulated than that in NT. The coexpressed genes of TUBA1B were enriched in the pathway of DNA replication, mismatch repair, cell cycle, pathogenic Escherichia coli infection, and spliceosome.

## Data Availability

The data used to support the findings of this study are included within the article.

## References

[1] Spreafico F., Ferrari A., Mascarin M. (2019). Wilms tumor, medulloblastoma, and rhabdomyosarcoma in adult patients: lessons learned from the pediatric experience. *Cancer Metastasis Reviews*.

[2] Fu W., Zhuo Z., Hua R.-X. (2019). Association of KRAS and NRAS gene polymorphisms with Wilms tumor risk: a four-center case-control study. *Aging*.

[3] Otto G. (2017). Kidney cancer: targeting Wilms tumour. *Nature Reviews. Nephrology*.

[4] Treger T. D., Chowdhury T., Pritchard-Jones K., Behjati S. (2019). The genetic changes of Wilms tumour. *Nature Reviews. Nephrology*.

[5] Zhu J., Jia W., Wu C., Fu W. (2018). Base Excision Repair Gene Polymorphisms and Wilms Tumor Susceptibility. *Base Excision Repair Gene Polymorphisms and Wilms Tumor Susceptibility*.

[6] Lin Z., Gasic I., Chandrasekaran V. (2020). TTC5 mediates autoregulation of tubulin via mRNA degradation. *Science*.

[7] Kim N. D., Park E. S., Kim Y. H. (2010). Structure-based virtual screening of novel tubulin inhibitors and their characterization as anti-mitotic agents. *Bioorganic & Medicinal Chemistry*.

[8] Reader J., Harper A. K., Legesse T. (2019). EP4 and Class III *β*-Tubulin Expression in Uterine Smooth Muscle Tumors: Implications for Prognosis and Treatment. *Cancers*.

[9] Cook A. D., Manka S. W., Wang S., Moores C. A., Atherton J. (2020). A microtubule RELION-based pipeline for cryo-EM image processing. *Journal of Structural Biology*.

[10] Tuszynski J. A., Friesen D., Freedman H. (2020). Microtubules as Sub-Cellular Memristors. *Scientific reports*.

[11] Consortium TG (2015). The Genotype-Tissue Expression (GTEx) pilot analysis: Multitissue gene regulation in humans. *The Genotype-Tissue Expression (GTEx) pilot analysis: multitissue gene regulation in humans*.

[12] Barrett T., Wilhite S. E., Ledoux P. (2012). NCBI GEO: archive for functional genomics data sets—update. *Nucleic Acids Research*.

[13] Kodama Y., Shumway M., Leinonen R., Collaboration INSD (2011). The Sequence Read Archive: explosive growth of sequencing data. *Nucleic Acids Research*.

[14] Rhodes D. R., Kalyana-Sundaram S., Mahavisno V. (2007). Oncomine 3.0: genes, pathways, and networks in a collection of 18,000 cancer gene expression profiles. *Neoplasia*.

[15] Athar A., Füllgrabe A., George N. (2019). ArrayExpress update – from bulk to single-cell expression data. *ArrayExpress update - from bulk to single-cell expression data*.

[16] Ritchie M. E., Phipson B., Wu D. (2015). limma powers differential expression analyses for RNA-sequencing and microarray studies. *Nucleic Acids Research*.

[17] Chen H., Boutros P. C. (2011). VennDiagram: a package for the generation of highly-customizable Venn and Euler diagrams in R. *BMC bioinformatics*.

[18] Liao Y., Wang J., Jaehnig E. J., Shi Z., Zhang B. (2019). WebGestalt 2019: gene set analysis toolkit with revamped UIs and APIs. *Nucleic Acids Research*.

[19] Dezső Z., Oestreicher J., Weaver A. (2014). Gene expression profiling reveals epithelial mesenchymal transition (EMT) genes can selectively differentiate eribulin sensitive breast cancer cells. *PLos One*.

[20] Yen T. J., Machlin P. S., Cleveland D. W. (1988). Autoregulated instability of *β*-tubulin mRNAs by recognition of the nascent amino terminus of *β*tubulin. *Nature*.

[21] Cleveland D. W. (1989). Autoregulated control of tubilin synthesis in animal cells. *Current opinion in cell biology*.

[22] Gasic I., Boswell S. A., Mitchison T. J. (2019). Tubulin mRNA stability is sensitive to change in microtubule dynamics caused by multiple physiological and toxic cues. *PLoS Biology*.

[23] Cheung C. H., Wu S. Y., Lee T. R. (2010). Cancer cells acquire mitotic drug resistance properties through beta I-tubulin mutations and alterations in the expression of beta-tubulin isotypes. *PLoS One*.

[24] Lu C., Zhang J., He S. (2013). Increased *α*-tubulin1b expression indicates poor prognosis and resistance to chemotherapy in hepatocellular carcinoma. *Digestive Diseases and Sciences*.

[25] Blenk S., Engelmann J. C., Pinkert S. (2008). Explorative data analysis of MCL reveals gene expression networks implicated in survival and prognosis supported by explorative CGH analysis. *BMC Cancer*.

[26] Pritchard-Jones K., Perotti D. (2019). WARNING: G-401 and SK-NEP-1 cell lines are not Wilms tumor cell lines. *Pediatric Blood & Cancer*.

[27] Stroup E. K., Budhipramono Y. Y. A., Hwang T. H. (2019). WT‐CLS1is a rhabdoid tumor cell line and can be inhibited bymiR‐16. *Cancer reports*.

